# Unmasking Multiple Myeloma: The Importance of Suspecting Malignancy in Atypical Chronic Pain Mimicking Degenerative Conditions—A Case Report

**DOI:** 10.1002/ccr3.70129

**Published:** 2025-01-16

**Authors:** Bi Mo, Sandra Sacks, Jerry Markar

**Affiliations:** ^1^ Los Angeles, David Geffen School of Medicine, Department of Anesthesiology and Perioperative Medicine, Division of Pain Medicine University of California Los Angeles California USA; ^2^ Los Angeles, David Geffen School of Medicine, Department of Internal Medicine, Division of Hematology‐Oncology University of California Los Angeles California USA

**Keywords:** atypical musculoskeletal pain, misdiagnosis of pathologic fractures, multiple myeloma, oncologic pain, osteolytic lesions, plasma cell malignancy

## Abstract

Heightened clinical vigilance for multiple myeloma is essential in patients presenting with atypical chronic pain progression. Symptoms may overlap with degenerative musculoskeletal conditions, frequently leading to misdiagnosis. This underscores the necessity of a thorough evaluation when symptoms are refractory to conventional therapies, in order to facilitate timely diagnosis and effective management of malignancy.

## 1 Introduction

Multiple myeloma (MM) is a hematologic malignancy characterized by the clonal proliferation of plasma cells in the bone marrow. These malignant plasma cells secrete monoclonal immunoglobulins (M‐proteins) or free light chains, leading to systemic complications such as hypercalcemia, renal dysfunction, anemia, and osteolytic bone lesions [1, 2, 3]. MM accounts for approximately 10% of all hematologic malignancies and predominantly affects older adults, with a median age at diagnosis of approximately 65 years [2].

One of the key features of MM is its impact on the skeletal system. Myeloma cells produce cytokines such as receptor activator of nuclear factor‐κB ligand (RANKL), which enhances osteoclastic activity while inhibiting osteoblastic function [1, 4, 5]. This imbalance leads to osteolytic lesions that cause pathological fractures and chronic bone pain, resulting in significant functional impairment [2, 6]. Unlike other malignancies that metastasize to bone, MM‐related bone disorder is distinct because of the absence of reactive bone formation [6].

The early presentation of MM often includes non‐specific symptoms such as chronic back/joint pain, fatigue, or localized bone discomfort, which are frequently misattributed to common musculoskeletal conditions like osteoporosis or degenerative joint disease [4, 7]. This symptom overlap can lead to misdiagnosis or delays in diagnosis, especially in older adults. In a study by Vijjhalwar et al. [4], more than 50% of the patients initially received other diagnoses, such as musculoskeletal disorders (47.8%), before MM was ultimately identified, highlighting the frequent misattribution of symptoms.

The diagnostic workup for MM requires a high index of suspicion, particularly in patients presenting with unexplained, persistent bone pain unresponsive to standard treatments. Radiographic imaging may reveal osteolytic lesions; however, these are often apparent only after significant disease progression, making early detection via routine X‐ray challenging [2].

In this report, we present the case of a patient who was ultimately diagnosed with MM following evaluation for chronic lower back pain and new‐onset shoulder pain initially attributed to age‐related degenerative conditions. This case underscores the diagnostic complexities of MM, emphasizing the importance of a comprehensive evaluation and maintaining a high index of suspicion for atypical presentations of musculoskeletal pain [6].

## 2 Case Presentation

### 2.1 Case History and Examination

A 66‐year‐old female with a significant medical history—including atrial fibrillation on chronic systemic anticoagulation, obesity (BMI > 35), type 2 diabetes mellitus managed with insulin and a glucagon‐like peptide‐1 (GLP‐1) receptor agonist, hypertension, and hyperlipidemia—presented for an interval evaluation after more than 12 months since her previous assessment. The patient reported progressive exacerbation of chronic, axial, non‐radiating lower back pain persisting for over 6 months, along with new bilateral shoulder pain over the preceding 2–3 months. Notably, she denied any antecedent trauma or specific precipitating event. The pain was rated as 6–8 out of 10 on a visual analog scale (VAS) and had markedly impaired her functional capacity, including activities of daily living such as reaching overhead. Despite evaluations by three orthopedic surgeons and treatment with lumbar epidural steroid injections and facet joint interventions from an external pain management specialist, she experienced no substantial symptomatic relief. Additionally, she reported a history of persistent abdominal pain over the past several months, which began prior to the onset of the lumbar and shoulder symptoms. A CT of the abdomen and pelvis revealed a 4 mm incidental pulmonary nodule at the right lung base, diffuse osteopenia, and multilevel degenerative changes involving the lumbar spine.

Interval laboratory results including comprehensive metabolic panel, lipid panel, and HgbA1c were within normal limits. Creatinine was 0.8 mg/dL (reference range: 0.6–1.3), estimated glomerular filtration rate (eGFR) was 78 mL/min/1.73 m^2^ (mildly decreased), and calcium was 10.1 mg/dL (reference range: 8.6–10.4).

On examination, the patient exhibited significant pain on range of motion of both shoulders, with tenderness along the anterior and posterior glenohumeral joints. Provocative tests, including passive resistance and the Neer impingement tests, were positive bilaterally. Gait assessment revealed an antalgic gait favoring the left side. Pain was elicited on palpation of lumbar facets and midline intervertebral disc regions, with positive loading tests of the lumbar spine bilaterally. The neurological exam was unremarkable, except for pain‐limited motor strength of the right lower extremity without focal motor or sensory deficits.

### 2.2 Investigations and Treatment

**FIGURE 1:**
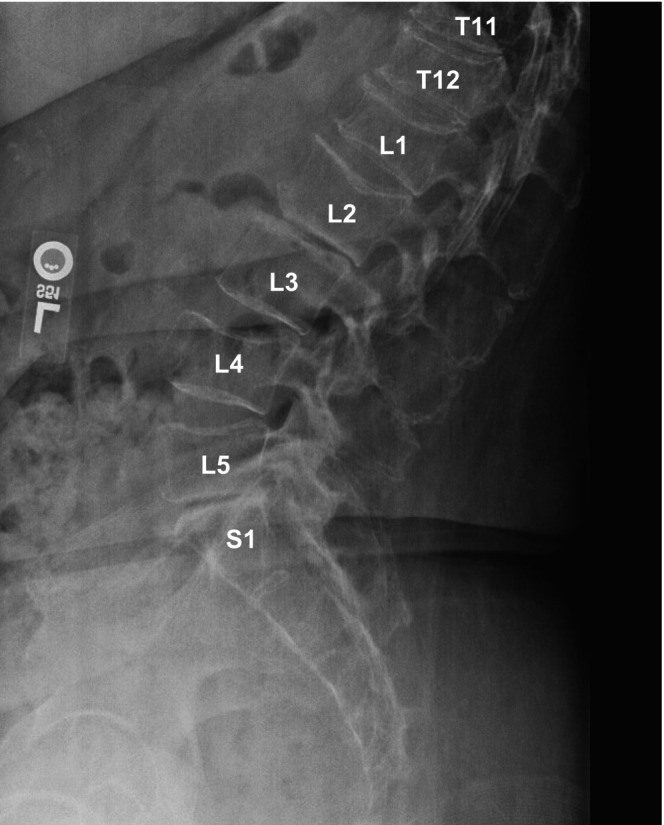
Lumbar X‐ray revealed multiple new vertebral body compression deformities involving T11, T12, L1, L2, L4, and L5.

**FIGURE 2:**
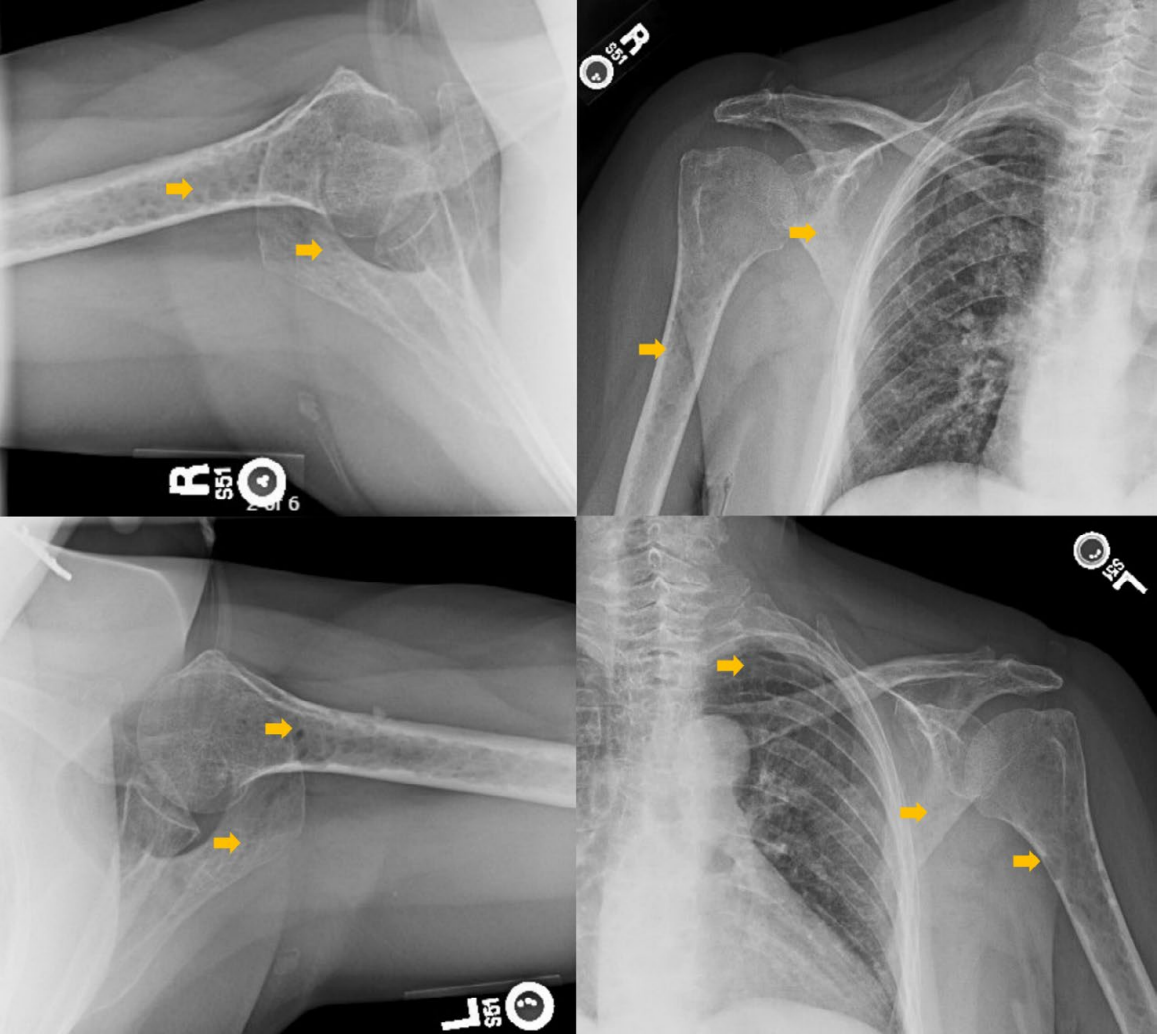
Bilateral shoulder X‐rays revealed innumerable osteolytic lesions (yellow arrow) involving the humerus, shoulder girdle bones, and left third rib.

**FIGURE 3:**
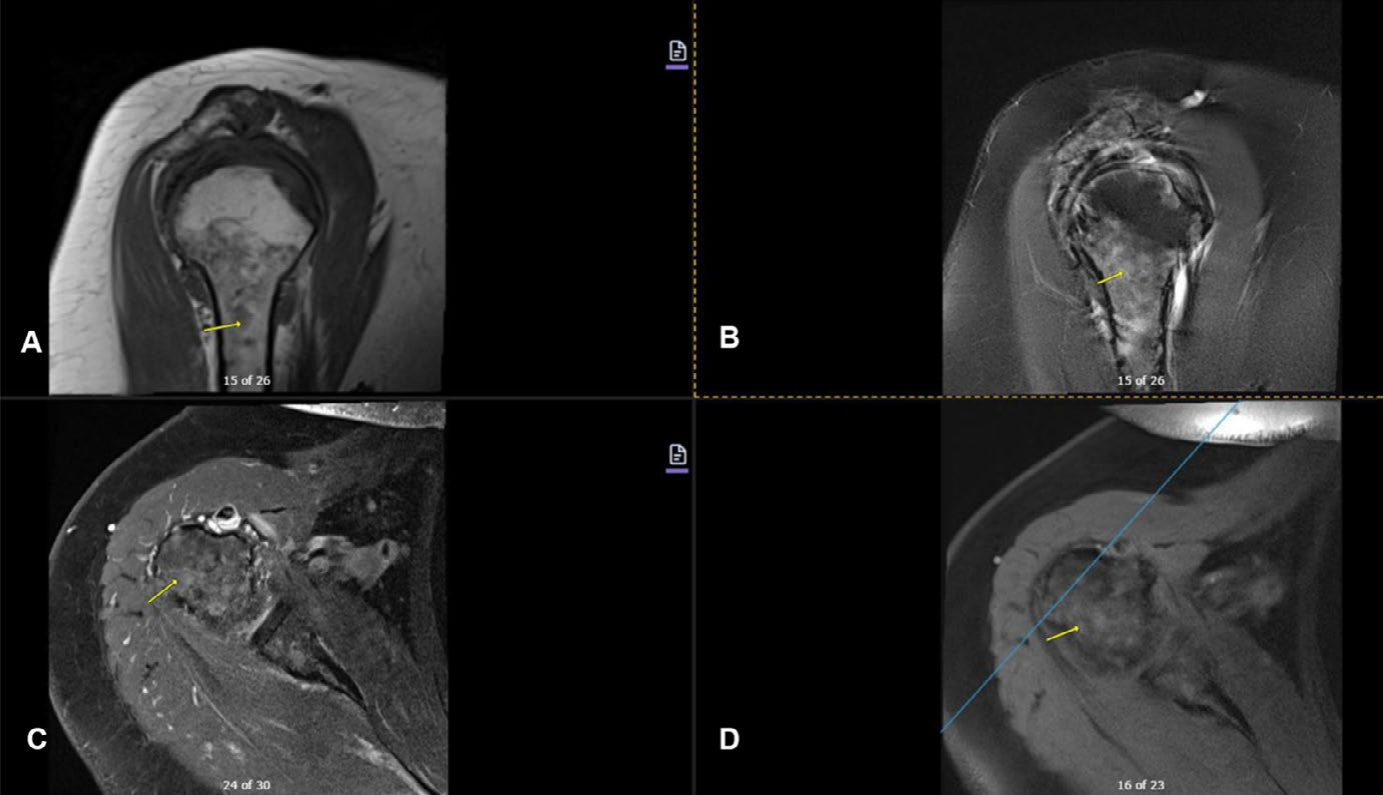
MRI of the right shoulder, with and without contrast, revealed innumerable infiltrative marrow‐replacing lesions (yellow arrow) throughout the shoulder. There was a full‐thickness, full‐width tear of the supraspinatus tendon, along with chronic strain at the myotendinous junction of the infraspinatus tendon. Additionally, an interstitial tear and tenosynovitis of the biceps tendon were identified. (A) Sagittal T1. (B) Sagittal T2 FS. (C) Axial T1 FS Post‐contrast. (D) Axial T1 FS Pre‐contrast.

**FIGURE 4:**
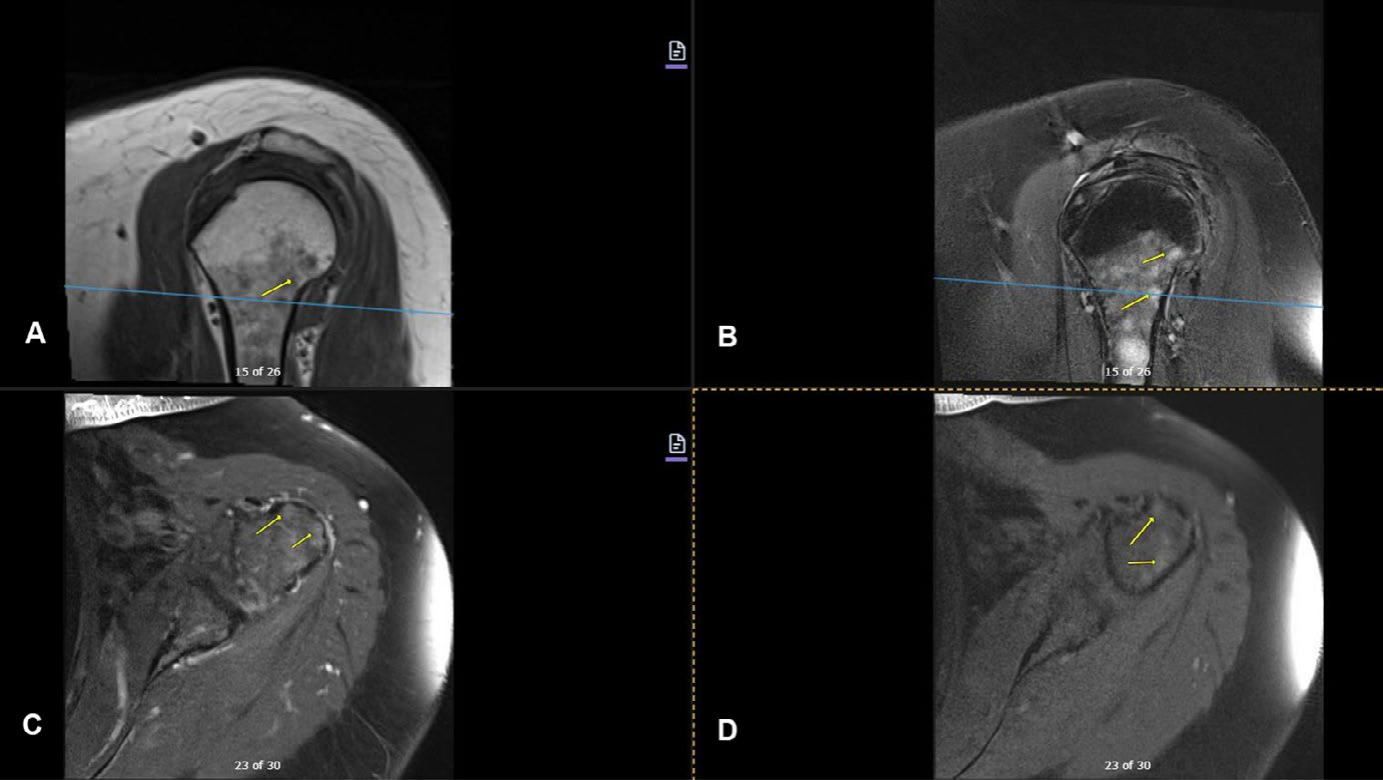
MRI of the left shoulder, with and without contrast, revealed innumerable infiltrative marrow‐replacing lesions (yellow arrow) throughout the shoulder. Left axillary lymphadenopathy was also noted. There was a full‐thickness tear of the anterior supraspinatus tendon in the setting of moderate tendinosis. Mild to moderate tendinosis of the infraspinatus and subscapularis was present. (A) Sagittal T1. (B) Sagittal T2 FS. (C) Axial T1 FS Post‐contrast. (D) Axial T1 FS Pre‐contrast.

**FIGURE 5:**
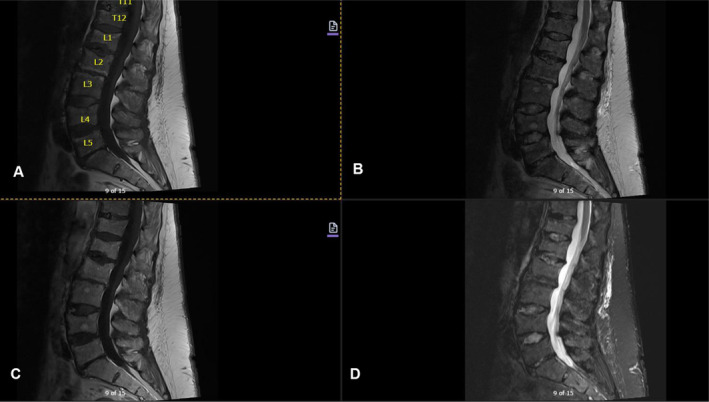
MRI of the lumbar spine, with and without contrast, revealed multiple chronic compression fractures throughout the lower thoracic and lumbar spine involving T11, T12, L1, L2, L4, and L5, resulting in 25%–50% height loss. No evidence of metastatic disease was found in the lumbar spine. (A) Sagittal T1 TSE. (B) Sagittal T2 TSE. (C) Sagittal T1 TSE Post‐contrast. (D) Sagittal IR.

Imaging studies conducted as part of the evaluation of the patient's symptoms, including X‐rays and MRIs, revealed significant findings highly suggestive of MM. The lumbar spine X‐ray (Figure 1) demonstrated osteopenia with multiple new vertebral compression deformities: T11‐L1 with mild to moderate height loss, L2 with mild height loss, and L4/L5 with mild height loss. Severe degenerative disc disease was noted at L2/L3 and L5/S1, and facet arthropathy predominantly in the lower lumbar region. There was also minimal retrolisthesis of L2 on L3 and anterolisthesis of L4 on L5 without dynamic instability. The shoulder X‐rays (Figure 2) revealed diffuse, ill‐defined lytic lesions throughout the shoulder girdle bones, highly suspicious for MM or possible metastasis. Additionally, bilateral rotator cuff pathology and left calcific tendinopathy at the pectoralis tendon insertion were noted. MRI of the shoulders (Figures 3 and 4) showed innumerable marrow‐replacing lesions with left axillary lymphadenopathy, along with bilateral full‐thickness supraspinatus tendon tears, moderate tendinosis of the left infraspinatus and subscapularis, and chronic strain of the right infraspinatus. The lumbar spine MRI (Figure 5) demonstrated multiple chronic compression fractures involving the lower thoracic and lumbar spine with 25%–50% height loss, without retropulsion of bone toward the spinal canal. Mild degenerative changes were also noted, most pronounced at L4‐5 and L5‐S1. There was no significant canal stenosis or evidence of metastatic disease. These findings prompted an urgent referral to oncology for further evaluation and management.

### 2.3 Outcome and Follow‐Up

**FIGURE 6:**
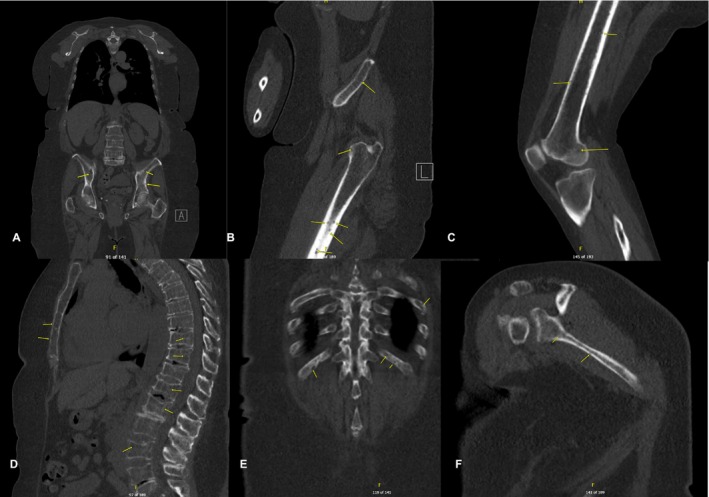
Whole‐Body CT myeloma workup revealed innumerable diffuse small lytic lesions (yellow arrow) throughout the axial and appendicular skeleton, which were highly concerning for multiple myeloma. (A) Bilateral pelvic girdle lesions. (B) Left femur and ilium lesions. (C) Right femur lesions. (D) Thoracic, lumbar spine, and sternum lesions. (E) Bilateral rib lesions. (F) Right scapular lesions.

An oncology workup was initiated, including whole‐body CT (WB‐CT) and MM laboratory panels (Table 1), following the correlation of the patient's history with imaging findings. This ultimately led to the definitive diagnosis of standard‐risk active MM. The WB‐CT myeloma workup (Figure 6) revealed numerous small lytic lesions distributed throughout both the axial and appendicular skeleton, findings that were highly concerning for MM, with osseous metastasis considered less likely. Additionally, multiple age‐indeterminate compression deformities were observed at T4, T6, and L1. A subacute pathologic fracture of the left third rib was also noted, along with scattered 3–4 mm calcified and noncalcified pulmonary nodules. These findings contributed to the overall assessment of the patient's disease status, further supporting the suspicion of MM. The bone marrow biopsy confirmed active MM with 60%–70% plasma cell infiltration, causing hypercellular marrow and reduced normal blood cell production. Immunohistochemistry showed plasma cells positive for CD138 with kappa light chain restriction, confirming their monoclonal nature. Flow cytometry detected a kappa‐restricted plasma cell population (4.4%) with no excess blasts or aberrant B‐ or T‐cells. Iron stains revealed decreased iron stores without ring sideroblasts. Cytogenetic analysis (FISH) identified a gain of chromosome 5, monosomy 13, and loss of 14q in 95% of plasma cells, abnormalities associated with standard‐risk myeloma according to the Mayo Stratification of Myeloma and Risk‐Adapted Therapy (mSMART 3.0). The oncology team initiated dexamethasone therapy at 20 mg weekly as the first step in treatment. Psychiatry and oncologic pain referrals were initiated as part of the integrative oncology strategy to support the patient's mental health and pain management throughout the treatment process.

**TABLE 1:** Multiple myeloma laboratory studies.

Component	Value	Reference range and units	Status
Hemoglobin	13.5 g/dL	11.2–15.7 g/dL	Normal
Prothrombin time	15.9 s	11.5–14.4 s	High
IgG, serum	664 mg/dL	700–1600 mg/dL	Low
IgM, serum	14 mg/dL	40–230 mg/dL	Low
IgA, serum	69 mg/dL	76–426 mg/dL	Low
Kappa quantitative free light chains, serum	10,063.29 mg/L	3.30–19.40 mg/L	High
Lambda quantitative free light chains, serum	3.90 mg/L	5.71–26.30 mg/L	Low
Kappa/Lambda free light chain ratio	2580.33	0.26–1.65	High
B‐2‐Microglobulin	3.9 mg/L	0.8–2.4 mg/L	High
Lactate dehydrogenase (LDH)	291 U/L	125–256 U/L	High
Free urinary lambda light chain	23.10 mg/L	0.00–3.79 mg/L	High
Lambda light chain, free, urine	21.16 mg/L	≤ 3.79 mg/L	High
Kappa light chain, free, urine	< 0.33 mg/L	≤ 32.90 mg/L	Normal

## 3 Discussion

Historically, the confirmation of active MM required detecting at least 10% clonal plasma cells in the bone marrow or identifying a biopsy‐proven plasmacytoma, along with evidence of myeloma‐defining events leading to organ damage, such as hypercalcemia, renal insufficiency, anemia, or bone lesions [1, 2]. Recently, the International Myeloma Working Group (IMWG) updated the diagnostic criteria for MM, marking a significant step forward in treatment planning. The revised criteria incorporate biomarkers validated through several independent studies, which predict an 80% or higher risk of myeloma‐related organ damage within 24 months [1].

The updated criteria also redefined myeloma‐defining events. Traditionally, these events included CRAB features, which consist of hypercalcemia (serum calcium levels above 0.25 mmol/L of the upper limit of normal or > 2.75 mmol/L, or 11 mg/dL), renal insufficiency (creatinine clearance below 40 mL/min or serum creatinine over 177 μmol/L, or 2 mg/dL), anemia (a hemoglobin concentration that is 20 g/L below the normal lower limit or less than 100 g/L, or 10 g/dL), and bone lesions (the presence of one or more osteolytic lesions on skeletal radiography, CT, or PET‐CT). In addition, the revised criteria now include new biomarkers, such as the presence of clonal plasma cells equal to or exceeding 60% on bone marrow examination, an involved/uninvolved serum free light chain (FLC) ratio of at least 100 (with the involved light chain level being 100 mg/L or greater), and more than one focal lesion of at least 5 mm in size on MRI [1].

MM presents a broad spectrum of clinical manifestations that overlap with benign conditions, making its diagnosis challenging [1, 2, 3, 4]. The clonal proliferation of malignant plasma cells results in systemic complications such as osteolytic bone disease, anemia, hypercalcemia, and renal dysfunction, necessitating a comprehensive diagnostic approach that includes clinical vigilance, imaging, and laboratory evaluation [1, 2, 4].

The heterogeneous presentation of MM often mimics benign musculoskeletal conditions, such as osteoarthritis, osteoporosis, or degenerative joint disease [4, 7]. Initial symptoms, including persistent bone pain, generalized fatigue, or localized discomfort, are frequently misattributed to common orthopedic issues, particularly in older adults. This misattribution can lead to delayed diagnosis, as seen in this case, where persistent lower back and shoulder pain were initially considered nonspecific musculoskeletal issues, leading to a delay in definitive diagnostic testing [1, 4].

Abdominal pain, although an uncommon early manifestation of MM, can complicate prompt diagnosis, particularly without the presence of classic CRAB features [7]. In this case, the patient initially presented with abdominal pain, prompting a CT scan of the abdomen and pelvis. The scan demonstrated no significant intra‐abdominal findings other than diffuse osteopenia of the axial skeleton. Four months later, the patient returned with new‐onset shoulder pain and worsening chronic lumbar spine pain that was refractory to treatment. Further gastroenterological evaluation attributed the abdominal pain to the potential use of nonsteroidal anti‐inflammatory drugs (NSAIDs) combined with a GLP‐1 receptor agonist, as well as gastroesophageal reflux disease. Proton pump inhibitor therapy was prescribed for symptom management, but the patient did not pursue the recommended esophagogastroduodenoscopy. Nevertheless, it remains important to consider that the abdominal pain may have been an early indication of MM, preceding the development of more pronounced skeletal involvement.

This case underscores the diagnostic challenges associated with the absence of classic MM features, which can hinder timely recognition of the disease. These challenges are compounded by the lack of consensus guidelines for standardized screening protocols when MM presents with symptoms mimicking benign conditions [1, 3, 4, 5, 7]. Initial imaging of this patient revealed diffuse osteopenia without lytic lesions, whereas lumbar spine radiographs demonstrated multilevel compression fractures without recent trauma, findings frequently misattributed to age‐related degenerative changes or postmenopausal osteogenic disorders [3]. The definitive diagnosis of MM was established only after a shoulder X‐ray revealed diffuse lytic bone lesions, leading to a WBCT scan using a myeloma‐specific protocol [6]. This highlights the importance of maintaining a high index of suspicion and utilizing appropriate imaging modalities for patients with persistent, unexplained pain that is refractory to conventional treatments. Similar cases have been reported by Yamane et al., where abdominal pain served as an initial manifestation of MM, emphasizing the broad spectrum of clinical presentations that can precede the more typical skeletal features of the disease [3, 4, 5, 7].

Interventional pain management specialists often encounter patients with chronic musculoskeletal pain that experience periodic exacerbations. It is imperative to recognize the potential for underlying oncologic etiologies when patients present with atypical, persistent pain that does not respond to conventional interventions, such as physical therapy, non‐opioid analgesics, or corticosteroid injections. In such cases, advanced imaging and further diagnostic testing are warranted to rule out underlying malignancies like MM [1, 3, 4, 5]. Timely diagnosis and referral to hematology–oncology can facilitate definitive care, ultimately improving outcomes for patients with undiagnosed malignancies [1, 6].

Therapeutic advancements in MM have transformed its management over the past few decades. Standard induction regimens often include proteasome inhibitors (e.g., bortezomib), immunomodulatory agents (e.g., lenalidomide), and corticosteroids, with autologous stem cell transplantation (ASCT) as a consolidative treatment for eligible patients [1, 8]. Maintenance therapy, typically using lenalidomide, helps prolong progression‐free survival, especially in patients who have undergone ASCT [1]. For those who are not candidates for transplantation due to age or comorbidities, combinations like daratumumab, lenalidomide, and dexamethasone (DRd) have demonstrated significant efficacy. Despite these advancements, MM remains incurable, with inevitable relapses, highlighting the ongoing need for innovation in treatment approaches that target the underlying disease biology more effectively [1, 8].

Cytogenetic analysis is crucial for risk stratification in MM and vital for guiding treatment [1, 2, 8]. High‐risk cytogenetic abnormalities, including chromosomal translocation t(4;14)(p16;q32), deletion of the short arm of chromosome 17, del(17p), and gain or amplification of chromosome arm 1q21 (1q21+), are linked to more aggressive disease and poorer prognosis [1, 8]. High‐risk cytogenetic abnormalities are also often linked to increased resistance to standard therapies [8]. In such cases, newer therapeutic agents, including chimeric antigen receptor T (CAR‐T) cell therapies and bispecific antibodies targeting the B‐cell maturing antigen (BCMA), have demonstrated promise, offering hope for patients refractory to conventional treatments [1, 8]. Nonetheless, the use of these novel therapies is limited by accessibility issues, high costs, and the need for specialized centers [1, 8].

Pain management is crucial in MM care, as bone disease significantly contributes to morbidity. Myeloma bone disease (MBD) results from disrupted bone remodeling, characterized by increased osteoclast activity leading to excessive bone resorption without adequate formation [2]. This imbalance causes lytic lesions, fractures, and severe pain, requiring a comprehensive management approach involving pharmacological and non‐pharmacological interventions [2, 5]. Bisphosphonates and denosumab are effective in reducing skeletal events through osteoclast inhibition, but pain control remains challenging, often requiring both opioid and non‐opioid analgesics along with adjunctive therapies [2, 5].

Integrative oncology supports MM patients, particularly in pain management and quality of life improvement. Complementary therapies like acupuncture, massage, and reflexology alleviate pain, reduce anxiety, and enhance well‐being, as endorsed by the Society for Integrative Oncology and the American Society of Clinical Oncology (ASCO) [9]. Such approaches are especially relevant given the chronic nature of MM and the prevalence of pain and emotional distress [9, 10].

MM's psychological burden, with anxiety and depression common but often undertreated, impacts patients' quality of life. The chronic, incurable nature of MM and treatment side effects significantly affect mental health. Integrating psychosocial support, such as cognitive behavioral therapy, mindfulness‐based stress reduction, and counseling, is crucial for enhancing well‐being, treatment adherence, and survival outcomes [10].

## 4 Conclusion

MM is a complex hematologic malignancy with heterogeneous clinical presentations, posing significant diagnostic and therapeutic challenges. This case highlights the critical need to maintain a high index of suspicion for MM in patients presenting with persistent, unexplained musculoskeletal symptoms, especially when conventional treatments fail to provide relief. While advances in treatment, including novel targeted therapies, offer hope for improved outcomes, MM remains incurable, necessitating ongoing research and individualized patient care strategies. Early diagnosis, standardized screening protocols, and a multidisciplinary approach involving specialists across hematology, oncology, radiology, and supportive care are essential for optimizing patient outcomes.

A thorough understanding of MM's pathophysiology, advancements in targeted therapies, and effective symptom management are key to prolonging survival and enhancing the patient experience. Interdisciplinary collaboration among healthcare professionals ensures timely diagnosis and comprehensive management of treatment regimens and symptoms. By integrating novel therapies, implementing supportive care measures, and addressing the multifaceted pain and psychological burden associated with MM, healthcare teams can significantly enhance quality of life, promote adherence to therapy, and provide hope for those confronting this challenging disease.

## Author Contributions

**Bi Mo:** conceptualization, data curation, formal analysis, investigation, validation, visualization, writing – original draft, writing – review and editing. **Sandra Sacks:** formal analysis, investigation, validation, writing – original draft, writing – review and editing. **Jerry Markar:** formal analysis, investigation, writing – original draft, writing – review and editing.

## Ethics Statement

Per UCLA IRB policy, a case report without identifying information does not require IRB approval.

## Consent

Written informed consent was obtained from the patient for the publication of this case report and any accompanying images. A copy of the written consent is available for review upon request by the editor(s).

## Conflicts of Interest

The authors declare no conflicts of interest.

## Data Availability

Data and materials utilized to prepare for this case report were drawn from the patient's electronic medical chart.
